# The relationship between soluble lymphocyte activation gene-3 and coronary artery disease

**DOI:** 10.3389/fcvm.2022.988582

**Published:** 2022-09-29

**Authors:** Xinlin Xiong, Zonggang Duan, Haiyan Zhou, Li Niu, Zhenhua Luo, Wei Li

**Affiliations:** ^1^Department of Cardiology, The Affiliated Hospital of Guizhou Medical University, Guiyang, China; ^2^Department of Central Laboratory, Guizhou Provincial People’s Hospital, The Affiliated People’s Hospital of Guizhou Medical University, Guiyang, China; ^3^Basic Medical College, Guizhou University School of Medicine, Guiyang, China

**Keywords:** lymphocyte activation gene-3, coronary artery disease, risk factor, immune, inflammation

## Abstract

**Background:**

Soluble lymphocyte activation gene 3 (sLAG3) may be used for diagnosis or prognosis in various diseases. However, the relationship between sLAG3 and coronary artery disease (CAD) are still unclear. This study aimed to investigate the levels of sLAG3 in patients with CAD, and its potential clinical association with the disease.

**Methods:**

A total of 66 subjects (49 patients with CAD and 17 control subjects without CAD) were enrolled. The sLAG3 level was measured using enzyme-linked immunosorbent assay (ELISA) kits. Clinical variables included demographics, biochemical markers, coronary angiography status, and ejection fraction of the heart (EF) were collected, and Gensini scores were calculated. LAG3 gene data was extracted from three datasets (GSE23561, GSE61144, GSE60993) in Gene Expression Omnibus (GEO) to compare differential expression between CAD and control subjects.

**Results:**

The sLAG3 level was significantly lower in the CAD vs. the controls (*P* < 0.05), and negatively associated with CAD [odds ratio (OR): 0.212, 95% confidential interval (CI): 0.060–0.746, *P* < 0.05]. Furthermore, the area under the curve (AUC) of sLAG3 level was significant (*P* < 0.05). The sLAG3 level in subjects with body mass index (BMI) ≥ 24 kg/m^2^ was lower compared to those with BMI < 24 kg/m^2^ (*P* < 0.05). The sLAG3 level was also negatively associated with BMI and diabetes mellitus (*P* < 0.05), though not associated with the Gensini scores or EF (*P* > 0.05). Lastly, the LAG3 gene expression in peripheral whole blood of patients with CAD were down-regulated compared to healthy controls (*P* < 0.05).

**Conclusion:**

The sLAG3 level was negatively associated with the occurrence but not severity of CAD. Meanwhile, the sLAG3 was negatively associated with BMI and diabetes mellitus, suggesting the reduced sLAG3 might be a novel risk factor for developing CAD.

## Introduction

Coronary artery disease (CAD), a chronic inflammatory immune disease, is one of the leading causes of heart failure and death globally (1, 2). It is characterized by plaque accumulation with the involvement of immune cells and cytokines (3, 4). The mechanism of CAD has been extensively researched. The underlying mechanism for initiation and progression of atherosclerotic plaques involves multifactorial gene expression, and the alteration of inflammatory factors (5, 6). Thus, the association between inflammation and CAD status is well established. Multiple studies have identified circulating biomarkers in peripheral blood for diagnosis, evaluation, or prognosis of CAD (7–10). For example, soluble suppression of tumorigenicity 2 was associated with all-cause mortality in patients with the chronic coronary syndrome (CCS) (7). Cystatin C may also be related to the severity of CAD in patients with diabetes mellitus (8). Lastly, red blood cell distribution width is independently associated with myocardial scar burden and left ventricular function in patients with CAD (9). These markers may provide significant therapeutic insight for treating CAD in clinical practice. Moreover, clinicians can greatly benefit from the discovery of novel markers to elucidate their understanding of disease progression.

Lymphocyte activation gene 3 (LAG3) is an immune checkpoint and transmembrane protein, which binds to major histocompatibility complex class II (MHC-II) and fibrinogen-like protein-1 (FGL1) as ligands for LAG3 (11–13). As a transmembrane protein, under the action of two metalloproteases (ADAM10, ADAM17), the extracellular segment of LAG3 can be released into the plasma to become soluble LAG3 (sLAG3) (14). It was reported that LAG3 was mainly expressed in lymphocytes including activated T cells and NK cells, which had been elaborated in tumors (15–18), hepatitis (19), and inflammatory bowel disease (20). Recently, a study found high expression of LAG3 on type 1 regulatory T cells (Tr1) in both patients with CAD and healthy participants (21). however, the expression correlated with disease severity as patients with three diseased vessels had significantly lower expression compared to those with only one diseased vessel, which indicated that LAG3 expression in Tr1 may be involved in CAD.

To date, the use of sLAG3 has been confirmed in the diagnosis or prognosis of a variety of diseases including cancer (22–24). Golden et al. found that the sLAG3 level could indicate a predisposition for CAD development (25). Nevertheless, the level of sLAG3 in the patients with CAD and the relationship between sLAG3 and CAD are still unclear. Therefore, in this study, we investigated the levels of sLAG3 in patients with CAD, and its potential clinical association with the disease.

## Patients and methods

### Participants

In this study, 66 subjects were enrolled from February 2021 to July 2021. Informed consent was obtained from all participants. All enrolled subjects were categorized into two groups: CAD (*n* = 49; 38 men and 11 women, mean age 63.61 ± 11.42 years) and control (*n* = 17; 9 men and 8 women, mean age 58.94 ± 9.37 years). The diagnostic criteria of CAD were based on literature (26–28). The patients with CAD had at least one main coronary artery stenosis with a luminal diameter of ≥ 50%, confirmed by coronary angiography. The inclusion criteria for patients with CAD were as follows: lumen stenosis of any major epicardial coronary artery 50% with or without clinical manifestation of ischemic symptoms, electrocardiogram changes, or cardiac troponins T changes (26). The control group consisted of healthy volunteers without atherosclerotic cardiovascular disease history or abnormal coronary angiography. Exclusion criteria included the presence of any of the following: malignant tumor, severe chronic heart failure, severe chronic liver and kidney disease, serious infection. Ethical approval for the research was got from the Ethics Committee of Guizhou Medical University Affiliated Hospital. This study was carried out in accordance with the Declaration of Helsinki.

### Gensini score of coronary artery stenosis

Gensini scores were adopted to quantify the severity of coronary artery lesion. The Gensini score is based on the extent and position of the stenosis of the coronary artery (29). The higher the score, the more severe the lesion.

### Detection of soluble lymphocyte activation gene 3 protein level and clinical data collection

Whole blood without anticoagulants was left for 2 h at room temperature or overnight at 4^°^C, followed by centrifugation for 15 min at 1,000 g. The supernatant was stored at −80^°^C until further analysis. The sLAG3 protein level was examined using enzyme-linked immunosorbent assay (ELISA) kits according to the manufacturer’s instruction. The ejection fraction of the heart (EF) and clinical baseline characteristics were collected during hospitalization. The clinical baseline characteristics included age, gender, body mass index (BMI), lipids levels, the history of smoking, hypertension, and diabetes mellitus.

### Lymphocyte activation gene 3 gene differential expression analysis in peripheral whole blood

Three datasets (GSE23561, GSE60993, and GSE61144) from the Gene Expression Omnibus (GEO) database^1^ were retrieved. The raw data were downloaded as MINiML files. The microarray data were normalized by the normalize quantiles function of the preprocessCore package in R software. Box plots were drawn using the “boxplot” R package, and the “ggord” package was used to draw the principal component analysis (PCA) plot. We extracted the LAG3 gene data from the three datasets and compared the difference in LAG3 gene expression in peripheral whole blood between the CAD and healthy control groups through the Wilcox test.

### Statistical analysis

Normally distributed variables are presented as mean ± standard deviation (SD), whereas non-normally distributed variables were expressed as medians with the interquartile range (IQR). Normally distributed variables were compared by student’s *t*-tests between the two groups. Non-normally distributed variables were compared by Mann-Whitney *U*-tests between the two groups. The categorical variables were expressed as percentages and compared by the chi-square test or Fisher’s exact test. Logistic regression analyses were performed to identify associations of sLAG3 with CAD in different regression models. The receiver operating characteristic (ROC) curve analysis was used to evaluate the area under the curve (AUC) of sLAG3 for discriminating CAD.

Spearman’s correlation analysis was applied to determine the correlation between sLAG3 and the variables. Multiple line regression analysis was applied to assess the independent association between sLAG3 and clinical variables. Statistical analysis was performed by SPSS v26.0. *P* < 0.05 was considered statistically significant.

## Results

### Clinical characteristics of subjects

Baseline clinical characteristics of the CAD and control groups are presented in Table 1. Hypertension and tobacco use were more prevalent in patients with CAD than in the control group (*P* < 0.05). The BMI of patients with CAD was higher compared to the control group (*P* < 0.05). There were no significant differences in age, gender, or history of diabetes between the patient with CAD and control groups (*P* > 0.05). The levels of triglyceride (TG), total cholesterol (TC), high-density lipoprotein cholesterol (HDL-C), and low-density lipoprotein cholesterol (LDL-C) were comparable between patients with CAD and the control group (*P* > 0.05).

**TABLE 1:** Clinical baseline characteristics of study participants.

Characteristics	CAD (*n* = 49)	Control (*n* = 17)	*P*
Age (years)	63.61 ± 11.42	58.94 ± 9.37	0.134
BMI (kg/m^2^)	24.90 ± 3.33	22.85 ± 2.81	0.027
Male (%)	38 (77.60%)	9 (52.90%)	0.105
Hypertension (%)	23 (46.9%)	3 (17.6%)	0.033
Diabetes mellitus (%)	13 (26.5%)	1 (5.90%)	0.147
Smoking (%)	30 (61.2%)	5 (29.4%)	0.024
TG (mmol/L)	1.47 (1.01, 2.03)	1.77 (0.99, 2.89)	0.253
TC (mmol/L)	4.17 ± 1.12	4.66 ± 0.85	0.105
HDL-C (mmol/L)	1.01 (0.84, 1.23)	1.05 (0.93, 1.44)	0.157
LDL-C (mmol/L)	2.71 ± 1.03	2.77 ± 0.90	0.842
ACS/CCS (*n*)	36/13		

CAD, coronary artery disease; ACS, acute coronary syndrome; CCS, chronic coronary syndrome; BMI, body mass index; TC, total cholesterol; TG, triglyceride; HDL-C, high-density lipoprotein cholesterol; LDL- C, low-density lipoprotein cholesterol.

### Reduced soluble lymphocyte activation gene 3 level in patients with coronary artery disease

**FIGURE 1:**
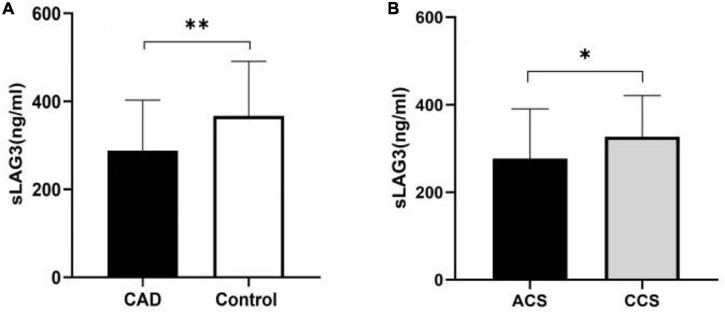
The level of the soluble lymphocyte activation gene-3 (sLAG3) in patients with coronary artery disease (CAD). **(A)** The sLAG3 was markedly reduced in patients with CAD. **(B)** The similar Level of sLAG3 between acute coronary syndrome (ACS) subgroup and chronic coronary syndrome (CCS) subgroup. The sLAG3 was compared using Mann-Whitney *U*-tests. ^**^P < 0.05, *P > 0.05.

We compared the sLAG3 level in patients with CAD and control subjects. As shown in Figure 1A, the sLAG3 level was significantly lower in patients with CAD than in the control group [288 (258.5–403) vs. 367 (323–491) ng/ml, *P* < 0.05]. Moreover, we divided patients with CAD into acute and CCS groups, and found no significant differences in sLAG3 protein level between the two groups (*P* > 0.05, Figure 1B).

### Correlation of soluble lymphocyte activation gene 3 level with coronary artery disease

The sLAG3 was transformed into dichotomous variables according to median of the sLAG3. Univariable analysis showed that the sLAG3 was inversely correlated to the occurrence of CAD [odds ratio (OR) = 0.212, *P* < 0.05]. After adjusting for age and sex, a significant negative association was observed between the sLAG3 level and CAD (OR = 0.143, *P* < 0.05). After adjusting for hypertension and BMI, the sLAG3 was inversely associated with CAD (OR = 0.221, *P* < 0.05). Additionally, after controlling for other variables including hypertension, diabetes, age, smoking, LDL-C, and HDL-C in different models, a lower sLAG3 level was also related to an increased risk of CAD (Table 2).

**TABLE 2:** The correlation of sLAG3 with coronary artery disease (CAD) in different models.

Variables	OR	95% CI	*P*
sLAG3	0.212	0.060–0.746	0.016
sLAG3 + Age + Sex	0.143	0.033–0.610	0.009
sLAG3 + Age + Smoking	0.196	0.046–0.830	0.027
sLAG3 + Hypertension + BMI	0.221	0.056–0.877	0.032
sLAG3 + Hypertension + Diabetes	0.211	0.056–0.802	0.022
sLAG3 + LDL-C + HDL-C	0.192	0.052–0.708	0.013

Multivariable logistic regression analyses were performed to identify the correlation of sLAG3 with CAD. The sLAG3 was transformed into dichotomous variables according to median of sLAG3. sLAG3, soluble lymphocyte activation gene-3; BMI, body mass index; HDL-C, high-density lipoprotein cholesterol; LDL-C, low-density lipoprotein cholesterol.

### Discriminative power of soluble lymphocyte activation gene 3 in patients with coronary artery disease

**FIGURE 2:**
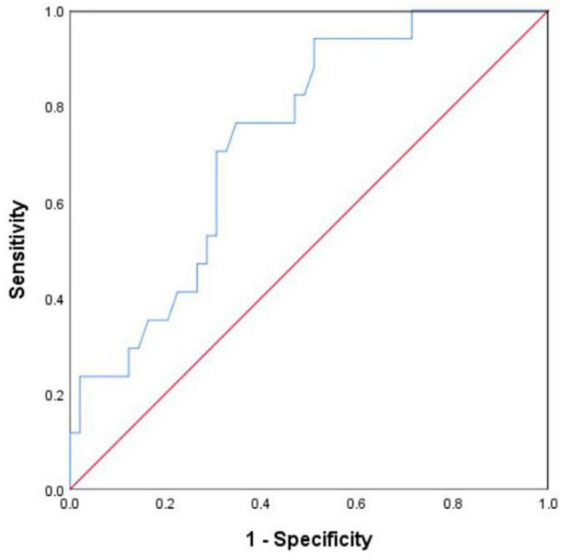
Receiver operating characteristic (ROC) curve of soluble lymphocyte activation gene- 3 (sLAG3) for identifying coronary artery disease.

The ROC curve was used to evaluate the diagnostic value of the sLAG3 level for CAD. As presented in Figure 2, the AUC value was 0.733 for CAD (*P* < 0.05), the cut-off value for sLAG3 was 280 ng/mL, whereas the corresponding sensitivity and specificity were 0.941 and 0.490, respectively.

### Soluble lymphocyte activation gene 3 is not associated with the progression of coronary artery lesions and cardiac function

We used Gensini scores to evaluate the severity of coronary artery lesions. Spearman’ s correlation analysis showed that the sLAG3 level did not correlate with the Gensini scores (*P* > 0.05). Spearman’s correlation analysis was used to determine the correlation between sLAG3 and EF from the ultrasound data of 43 subjects. There was no significant correlation between the sLAG3 level and EF (*P* > 0.05).

### Association of soluble lymphocyte activation gene 3 level with body mass index and diabetes mellitus

**FIGURE 3:**
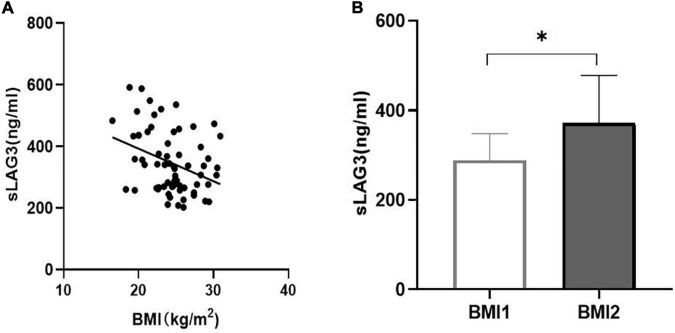
The correlation between soluble lymphocyte activation gene-3 (sLAG3) and body mass index (BMI). **(A)** sLAG3 was negatively correlated with BMI (*r* = −0.314, *P* < 0.05). **(B)** The sLAG3 level according to BMI 24 kg/m^2^, BMI1 ≥ 24 kg/m^2^, BMI2 < 24 kg/m^2^. sLAG3 between two groups was compared using Mann-Whitney *U*-tests. *P < 0.05.

We analyzed the correlation of sLAG3 with risk factors for CAD, and found a negative correlation between sLAG3 and BMI (*P* < 0.05, Figure 3A). All subjects were classified into two groups based on their BMI 24 kg/m^2^: BMI1 ≥ 24 kg/m^2^ and BMI2 < 24 kg/m^2^. The level of sLAG3 in BMI1 subjects was lower compared to BMI2 subjects (*P* < 0.05, Figure 3B). After additional consideration of independent variables, including sex, age, BMI, hypertension, diabetes, smoking, LDL-C, and HDL- C, multivariable regression analysis revealed an independent association of sLAG3 with BMI and diabetes mellitus (see Table 3). However, we did not observe the association between sLAG3 with other clinical variables including the lipid profiles (TG, TC, HDL-C, LDL-C), age, hypertension, smoking, and sex.

**TABLE 3:** The association of sLAG3 with BMI and diabetes mellitus.

Variables	Standardized coefficient β	*P*
BMI	−0.448	0.002
Diabetes mellitus	−0.260	0.042

Multivariable regression analysis were performed to identify the association of sLAG3 with BMI and diabetes mellitus. The sLAG3 was dependent variable; the independent variables included sex, age, BMI, hypertension, diabetes, smoking, high-density lipoprotein cholesterol, low-density lipoprotein cholesterol. sLAG3, soluble lymphocyte activation gene-3; BMI, body mass index.

### Reduced expression of lymphocyte activation gene 3 gene of peripheral blood of patients with coronary artery disease

**FIGURE 4:**
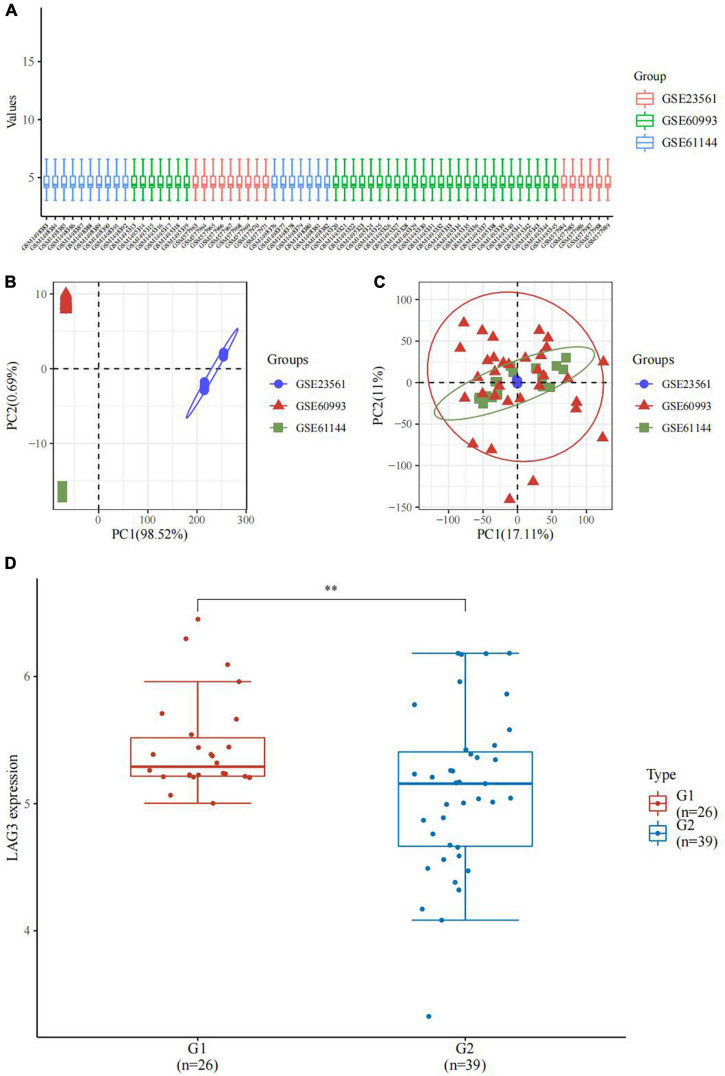
LAG3 gene in coronary artery disease (CAD). **(A)** The box plot of the normalized data. Different colors represent different datasets. Rows represent samples, and columns represent the gene expression values in the samples. **(B)** Principal component analysis (PCA) results before batch removal for three data sets. Different colors represent different data sets. As shown in the schematic diagram, the three data sets are separated without any intersection. **(C)** PCA results after batch removal. The schematic diagram shows the intersection of three data sets which can be used as a batch of data for subsequent analysis. **(D)** The expression distribution of the LAG3 gene in peripheral blood. The horizontal axis represents different groups of samples: G1, normal control; G2, CAD group. ^**^P < 0.05.

We retrieved LAG3 gene expression data from three datasets in GEO database containing patients with CAD (*n* = 39) and healthy controls (*n* = 26). We found that LAG3 gene expression in the peripheral blood of patients with CAD was down-regulated compared to the control group (*P* < 0.05, Figures 4A–D).

## Discussion

We revealed that sLAG3 level is reduced in CAD, accompanied by down-regulation of LAG3 gene in peripheral blood. Moreover, the sLAG3 level was negatively associated with BMI and diabetes mellitus. LAG3 exists mainly in two forms: transmembrane-cytoplasmic domains and extracellular domains (30). As a transmembrane protein, the expression of LAG3 in lymphocytes potentially has a negative immunomodulatory effect on immune cells (12, 31–34). In patients with systemic lupus erythematosus (SLE), human immunodeficiency virus (HIV), or carotid artery stenosis, membrane LAG3 expression was also associated with the disease activity and disease progression (35–37). sLAG3 is formed after part of the transmembrane protein of LAG3 is removed by ADAM10 and ADAM17 (14, 38). The level and function of sLAG3 have been investigated in certain diseases. Studies have shown that sLAG3 was reduced in patients with gastric cancer with significant diagnostic and prognostic potential (22), and increased in non-small cell lung cancer (39). Furthermore, sLAG3 level has also been shown to be progressively decreased with the severity of lung cancer stages (40). Another study found that sLAG3 level was decreased in patients suffering from severe aplastic anemia (24). In the current study, we revealed that the sLAG3 level was reduced in patients with CAD compared to the control group, and a lower sLAG3 level was associated with CAD. Furthermore, the sLAG3 level by itself could discriminate CAD from normal controls with high sensitivity. Collectively, our findings indicated that sLAG3 correlated with the occurrence of CAD. Our findings for reduced LAG3 in patients with CAD were similar to previous reports by Golden et al. (25), that found initial plasma sLAG3 levels were lower in patients suffering from CAD during follow-up compared to those without CAD, and negatively correlated with the occurrence of future CAD. Nevertheless, the physiological function of sLAG3 remains unclear. Li et al. reported that sLAG3 might be a byproduct without physiological function produced on the T cell surface (14). However, evidence from another study also showed that differentiation of macrophages and dendritic cells was negatively regulated by sLAG3, which could attenuate the antigen-presentation function of dendritic cells (41). In the Multi-Ethnic Study of Atherosclerosis (MESA) (25), the sLAG3 level of participants was positively correlated with anti-inflammatory cytokine IL-10 level. These above mentioned findings indicated that decreased sLAG3 levels may be associated with pro-inflammatory effects in CAD or atherosclerosis. However, further investigation of the *in vivo* effects of sLAG3 in atherosclerosis is required.

Obesity is well known as an important risk factor for CAD. For instance, a higher BMI increases the risk of developing CAD and type 2 diabetes (42, 43). Diabetes mellitus is also a major contributor to CAD (44). After controlling for variables, the sLAG3 was independently associated with BMI and diabetes mellitus. These results suggested that the reduced sLAG3 level could be a novel risk factor for CAD.

Our results demonstrated no association between sLAG3 levels and Gensini scores, suggesting that sLAG3 did not correlate with the severity of coronary artery lesions in CAD. Besides, we also did not find an association of sLAG3 with EF in the current study. This finding appeared to be supported by a report by Pallikkuth et al. (36). The reason for the decrease of sLAG3 in patients with CAD is still unknown. Many factors such as race, and inflammatory factors may influence the sLAG3 level (25, 45). Besides, LAG3 expression may be also regulated by methylation, T cell receptor pathway activation, cytokines, and metalloproteinase (46). Additionally, The genotype of lipoprotein scavenger receptor BI (SCARB1) rs10846744 associated with atherosclerosis and atherosclerotic cardiovascular disease was also related to alteration of sLAG3 level (25), suggesting that sLAG3 level was susceptible to alteration of gene. In the present study, by analyzing LAG3 gene expression data from three GEO datasets, we found LAG3 gene expression was down-regulated in the peripheral blood of patients with CAD. Meanwhile, sLAG3 was reduced in CAD, in alignment with the down-regulation of LAG3 gene expression in peripheral blood, suggesting reduced LAG3 gene expression may contribute to reduction of sLAG3 in peripheral blood in CAD. In addition, the present study indicated that both diabetes mellitus and higher BMI could have an independent impact on reducing the sLAG3 level.

However, as this was a cross-sectional exploratory study, the mechanism by which sLAG3 decreased in patients with CAD needs to be further researched. The association of sLAG3 and LAG3 gene with CAD indicates LAG3 regulation as a potential therapeutic target for CAD.

Several important limitations need to be highlighted in this study. First, the sample size was relatively small, although the sample size met the statistical requirements, as a result of the sample size and conditions of logistic regression, we only used a combination of three variables to investigate the association of sLAG3 with CAD between different models by logistic regression. Moreover, we did not assess the difference among LAG3 proteins including total protein, membrane proteins, and intracellular protein levels of lymphocytes in peripheral blood of the CAD and control groups. Whether these proteins levels of LAG3 were changed may improve our understanding of LAG3 function in CAD. In addition, the mechanism of sLAG3 in CAD has not been elucidated. Therefore, future research needs to explore the pathophysiological mechanism of LAG3 in immune cells in both CAD and atherosclerosis. Finally, the causal relationship of alteration of LAG3 protein and gene expression with CAD should be investigated in the future.

## Conclusion

Our study demonstrates that sLAG3 level is reduced in patients with CAD, accompanied by down-regulation of LAG3 gene in peripheral blood. The reduced sLAG3 correlated with the occurrence of CAD. Meanwhile, the reduced sLAG3 might be a novel risk factor for CAD. The sLAG3 level was not associated with the severity of CAD. The definitive role of sLAG3 in CAD requires further investigation in future experiments.

## Data availability statement

The datasets presented in this study can be found in online repositories. The names of the repository/repositories and accession number(s) can be found below: https://www.ncbi.nlm.nih.gov/geo/, GSE23561, GSE60993, and GSE61144.

## Ethics statement

The studies involving human participants were reviewed and approved by the Ethics Committee of Guizhou Medical University Affiliated Hospital. The patients/participants provided their written informed consent to participate in this study.

## Author contributions

XX and HZ draft the manuscript. XX and ZD carried out the statistical analysis. LN, XX, and ZD participated in data collection and interpretation of results. ZL and WL contributed to discussion, edited the manuscript, designed, and supervised the project. All authors contributed to the article and approved the submitted version.

## Footnotes

1
https://www.ncbi.nlm.nih.gov/geo/
